# Early versus delayed enteral nutrition in ICU patients with sepsis: a propensity score-matched analysis based on the MIMIC-IV database

**DOI:** 10.3389/fnut.2024.1370472

**Published:** 2024-06-24

**Authors:** Fuchao Xu, Jianxin Xu, Jinjin Ma, Wenbo Xu, Shuangshuang Gu, Geng Lu, Jun Wang

**Affiliations:** ^1^Department of Emergency Medicine, Nanjing Drum Tower Hospital, Nanjing, China; ^2^Department of Emergency Medicine, Drum Tower Hospital, Nanjing Medical University, Nanjing, China; ^3^Department of Rehabilitation Medicine, First Affiliated Hospital of Sun Yat-sen University, Guangzhou, China

**Keywords:** enteral nutrition, Sepsis, propensity score, MICU, SICU, MIMIC-IV

## Abstract

**Background:**

Early enteral nutrition (EN) is recommended for sepsis management, but its optimal timing and clinical benefits remain uncertain. This study evaluates whether early EN improves outcomes compared to delayed EN in patients with sepsis.

**Methods:**

We analyzed data of septic patients from the MIMIC-IV 2.2 database, focusing on those in the Medical Intensive Care Unit (MICU) and Surgical Intensive Care Unit (SICU). Patients who initiated EN within 3 days were classified into the early EN group, while those who started EN between 3 and 7 days were classified into the delayed EN group. Propensity score matching was used to compare outcomes between the groups.

**Results:**

Among 1,111 patients, 786 (70.7%) were in the early EN group and 325 (29.3%) were in the delayed EN group. Before propensity score matching, the early EN group demonstrated lower mortality (crude OR = 0.694; 95% CI: 0.514–0.936; *p* = 0.018) and shorter ICU stays (8.3 [5.2, 12.3] vs. 10.0 [7.5, 14.2] days; *p* < 0.001). After matching, no significant difference in mortality was observed. However, the early EN group had shorter ICU stays (8.3 [5.2, 12.4] vs. 10.1 [7.5, 14.2] days; *p* < 0.001) and a lower incidence of AKI stage 3 (49.3% vs. 55.5%; *p* = 0.030). Subgroup analysis revealed that early EN significantly reduced the 28-day mortality rate in sepsis patients with lactate levels ≤4 mmol/L, with an adjusted odds ratio (aOR) of 0.579 (95% CI: 0.361, 0.930; *p* = 0.024).

**Conclusion:**

Early enteral nutrition may not significantly reduce overall mortality in sepsis patients but may shorten ICU stays and decrease the incidence of AKI stage 3. Further research is needed to identify specific patient characteristics that benefit most from early EN.

## 1 Background

Sepsis is the dysregulated immune response to infection that leads to life-threatening organ dysfunction (1, 2). Sepsis affects nearly 50 million people worldwide each year and causes approximately 11 million deaths (2). The mortality rate for patients with sepsis who were treated in the intensive care unit was as high as 41.9% (3). Sepsis mortality rates have decreased in recent years as a result of published sepsis guidelines, advances in medical technology, and antibiotic therapy, but rates remain high (4–9).

In recent years, the role of nutrition in critical illness has received increasing attention (10). In critically ill adults, early administration of nutrients has been associated with improved clinical outcomes (11). Recent research has confirmed that there is dysbiosis of the gut microbiota in patients with sepsis (12). The association between disease and disturbances in the gut microbiome has been demonstrated to result in clinical deterioration and the development of multiple organ dysfunction syndrome (MODS) (13). Therefore, timely enteral nutrition supplementation is also a crucial aspect of treating sepsis patients (14). Preclinical studies have demonstrated that earlier enteral nutrition can protect human intestinal epithelial barrier function (15, 16). For hospitalized patients, early enteral nutrition may be beneficial (17). A recent study found that early administration of enteral nutrition reduces the incidence of ventilator-associated pneumonia in patients with severe trauma who require invasive ventilation (18). The early administration of enteral nutrition in patients with sepsis and septic shock has potential physiologic advantages related to the maintenance of gut integrity and prevention of intestinal permeability, dampening of the inflammatory response, and modulation of metabolic responses that may reduce insulin resistance (19, 20). However, the benefit of early enteral nutrition for sepsis patients remains controversial. Early nutrition support in the intensive care unit has been found to be significantly associated with higher 28-day mortality rates, particularly in younger patients with less severe illness (21, 22). Recent studies have shown neither a direct benefit nor harm from early enteral nutrition in sepsis patients (23). In the most recent sepsis survivor exercise guidelines, a weak recommendation has been made that suggests early enteral nutrition therapy for sepsis patients. However, there is insufficient evidence to support this recommendation (9). The timing and benefits of enteral nutrition in patients with sepsis in the intensive care unit (ICU) have not been clearly established. In this study, we aimed to assess the potential benefits of early enteral nutrition in comparison to delayed enteral nutrition. To test this hypothesis, we conducted a retrospective cohort study utilizing the MIMIC-IV 2.2 database.

## 2 Materials and methods

### 2.1 Overview

This study was a retrospective observational study utilizing the Medical Information Mart for Intensive Care IV database. (MIMIC-IV version 2.2 was most recently updated on January 5, 2023) (24). The MIMIC-IV is a large, single-center database that contains data on patients who were admitted to the intensive care unit (ICU) of a large tertiary care hospital in Boston. The database contains 73,181 hospital admissions for adult patients (18 years of age and older) who were admitted to the intensive care unit from 2008 to 2019.

All information in this database has been deidentified, making it impossible to identify individual patients. As a result, this study is not classified as human subject research and does not require consent from the patients owing to unidentified health information. The MIMIC-IV 2.2 database’s creation was approved by institutional review boards at both the Massachusetts Institute of Technology (MIT, Cambridge, MA) and BIDMC. The author, Fuchao Xu, was granted access to the MIMIC-IV 2.2 database after completing the Human Subject Research course (Certification Number: 52712098).

### 2.2 Selection of participants

Patients over 18 years of age with a sepsis diagnosis who received enteral nutrition during their stay in the intensive care unit were considered for inclusion. The sepsis diagnoses complied with the definition of sepsis 3.0 (1). We analyzed only the data from the initial admission to the Medical Intensive Care Unit (MICU) and Surgical Intensive Care Unit (SICU) for each patient. Patients who spent less than 72 h in the ICU were excluded.

In our study, we collected objective patient information, including age, sex, body mass index (BMI), care unit, and race. Additionally, we recorded vital signs taken within the first 24 h of ICU admission, specifically heart rate, respiratory rate, mean arterial pressure, temperature, glucose levels, use of vasopressors, and total urine output during the first 24 h. The laboratory results included pH, arterial oxygen partial pressure (PO2), arterial carbon dioxide partial pressure (PCO2), Pao2/Fio2 ratio, lactate, white blood cell (WBC) count, hemoglobin levels, platelet count, creatinine, blood urea nitrogen, serum albumin, and blood electrolyte levels (chlorine, calcium, potassium, sodium). For variables recorded multiple times during the first 24 h, we selected the values related to the greatest disease severity. Interventions within the initial 24 h comprised invasive mechanical ventilation, continuous renal replacement therapy, invasive arterial pressure monitoring, and a peripherally inserted central catheter. The severity scores during the first day of admission into the ICU were evaluated by the Acute Physiology Score III (APS III), Oxford Acute Severity of Illness Score (OASIS), Logistic Organ Dysfunction System (LODS), Sequential Organ Failure Assessment (SOFA), and Charlson Comorbidity Index (CCI). Comorbidities, such as congestive heart failure, chronic pulmonary disease, mild liver disease, diabetes, chronic kidney disease, and cancer, were recorded. The data extraction code is available on GitHub.^1^ PostgreSQL tools (version 15) were used for all data extractions (25).

**Figure 1:**
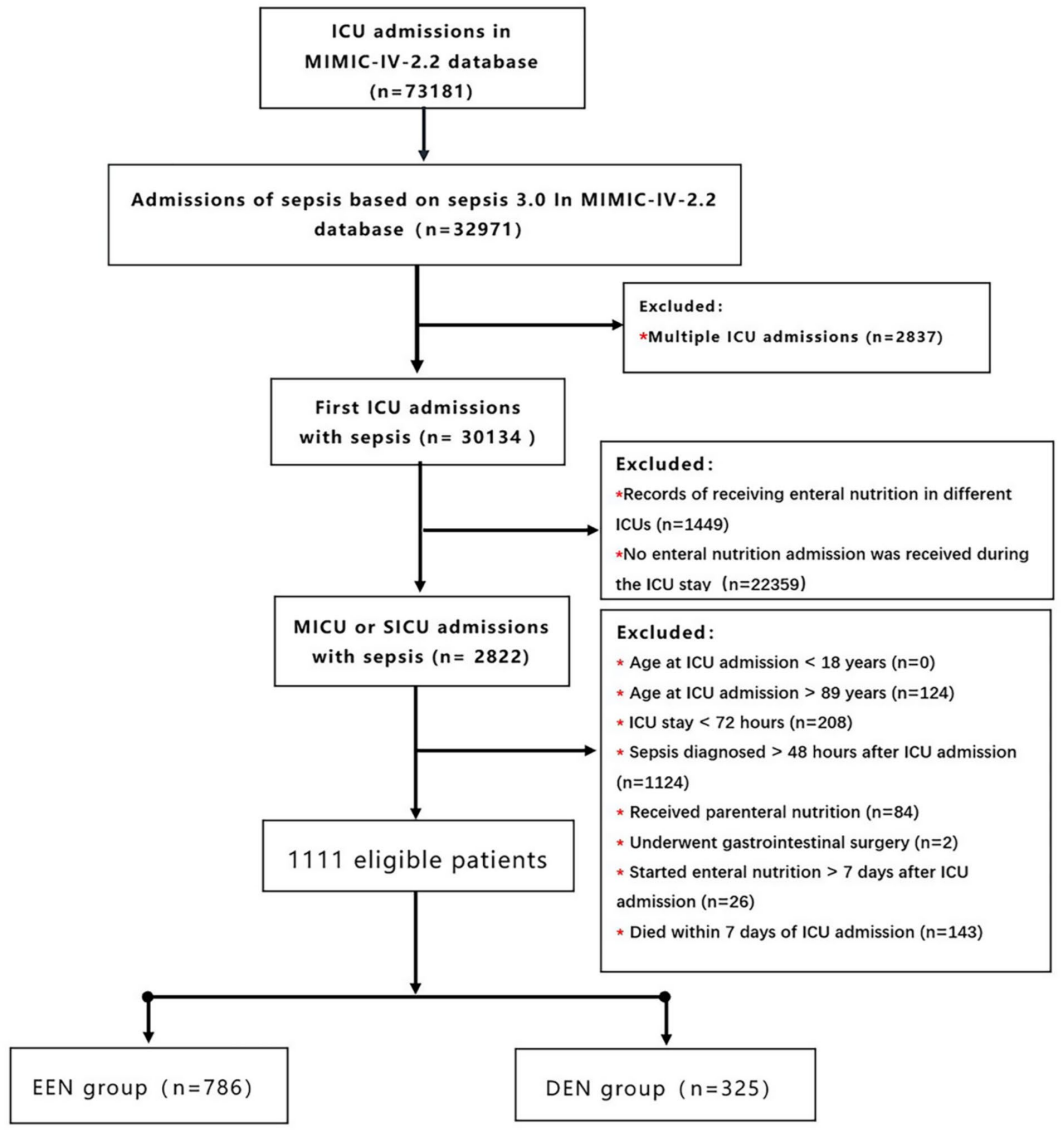
Flow chart of participant selection. MIMIC-IV, medical information mart for intensive care IV; ICU, intensive care unit; EEN, early enteral nutrition; DEN, delayed enteral nutrition.

We included only patients with sepsis who were admitted to the MICU or SICU for the first time. The patient flow diagram is presented in Figure 1. The following exclusion criteria were applied: (1) received enteral nutrition in different ICUs; (2) no enteral nutrition admission during the ICU stay; (3) patients admitted to a care unit other than the medical ICU and surgical ICU; (4) age at ICU admission <18 years; (5) age at ICU admission >89 years (for all patients older than 89 years, the database was adjusted for their age, so we excluded them); (6) ICU stay <72 h; (7) Sepsis diagnosed >48 h after ICU admission; (8) Received parenteral nutrition; (9) Underwent gastrointestinal surgery. (We examined whether septic patients underwent gastrectomy (ICD-9-CM codes 43.5–43.9, ICD-10-PCS codes 0DB60ZZ, 0DB70ZZ), bowel resection (ICD-9-CM codes 45.7x, 45.8x, ICD-10-PCS codes 0DBB0ZZ, 0DBC0ZZ), or gastrointestinal anastomosis (ICD-9-CM codes 44.x) procedures based on their respective diagnostic codes). (10) Started enteral nutrition >7 days after ICU admission; and (11) died within 7 days after ICU admission (to avoid immortal time bias).

### 2.3 Group assignment

By the newly published guidelines for sepsis and nutrition guidelines for critically ill patients. We divided the patients who started EN within 3 d after ICU admission into the early EN group and those who started EN 3–7 d after ICU admission into the delayed EN group (9, 26–29).

### 2.4 Statistical analysis

We employed the Kolmogorov–Smirnov test and the Shapiro–Wilk test to analyze continuous variables for normal distribution. Continuous variables are represented using either the mean ± standard deviation or the median (interquartile range), depending on their distribution. Categorical variables are presented as proportions. Appropriate statistical tests, such as the t test, analysis of variance, and the Mann–Whitney U test, were used for comparisons. The χ2 test was used for comparing categorical variables.

A propensity score–matching method was applied to compare the outcomes between the EEN and DEN groups (30). Propensity score matching (PSM) was performed to balance the baseline characteristics between the EEN group and the DEN group, so we used a logistic regression model to calculate the propensity score for each patient. In our study, 1:1 nearest neighbor matching was applied with a caliper width of 0.05. After PSM, standardized mean differences (SMDs) were used to evaluate the balance of characteristics between the two groups. A variable can be considered imbalanced between groups if its SMD is greater than 0.1 (31, 32). The implementation of the propensity-matching analysis is in R (version 4.3.1).

The primary outcome was 28-day mortality. Secondary outcomes included 60-day mortality, ICU mortality, length of stay in the ICU (LOS ICU), and the incidence of stage AKI. Kaplan–Meier curves were used to compare the survival of the two groups at 28 and 60 days before and after the propensity-matching procedure. Subgroup analysis was used to identify the specific population that may be more likely to benefit from EEN. We performed subgroup analyses according to age, sex, type of ICU, SOFA score, and lactate. Single-factor logistic analysis and multifactor logistic analysis were used to evaluate the association between early enteral nutrition and 28-day mortality in different subgroups. All subgroup results are presented in a forest plot. For missing data, utilize the MICE package in R to perform multiple imputation on the dataset (33) (Supplementary Figure S1). All the statistical analyses were carried out using R (version 4.3.1), and a *p* value of <0.05 (two-sided) was used to indicate statistical significance.

## 3 Results

### 3.1 Demographic data and baseline characteristics

The MIMIC-IV database included 32,971 adult patients diagnosed with sepsis, from which our study cohort comprised 1,111 patients (Figure 1). Among these, 786 (70.7%) patients were classified into the early enteral nutrition (EEN) group, initiating enteral nutrition within 3 days after admission, while 325 (29.3%) patients were assigned to the delayed enteral nutrition (DEN) group, commencing enteral nutrition between 3 and 7 days after admission. Characteristics of patients in the EEN and DEN groups are summarized in Table 1. Notably, patients in the DEN group demonstrated significantly higher lactate levels (2.80 [1.70, 4.60] vs. 2.20 [1.50, 3.69]; *p*<0.001) and elevated disease severity scores, including LODS score (7.00 [6.00, 10.00] vs. 7.00 [5.00, 9.00]; *p*<0.001), and APS III score (66.00 [51.00, 82.00] vs. 57.50 [44.00, 74.00]; *p*<0.001) compared to the EEN group. Within the initial 24 h following ICU admission, the EEN group exhibited a higher likelihood of requiring invasive mechanical ventilation (631 (80.3%) vs. 226 (69.5%); *p*<0.001) and first-day urine output (1382.50 [770.50, 2233.75] vs. 1113.00 [455.00, 1955.00]; *p*<0.001).

**Table 1:** Clinical characteristics of patients before and after propensity score matching.

	Before PSM			After PSM		
Variables	Early EN (*n* = 786)	Delayed EN (*n* = 325)	*p*-value	Early EN (*n* = 290)	Delayed EN (*n* = 290)	*p*-value
**Age (years)**	64.23 [52.88, 74.45]	62.30 [51.62, 73.59]	0.198	64.13 [51.76, 73.08]	63.27 [52.16, 73.94]	0.908
**Male**	412 (52.4)	191 (58.8)	0.062	169 (58.3)	164 (56.6)	0.737
**BMI (kg/m** ^**2**^ **)**	28.10 [24.26, 34.03]	28.83 [24.56, 34.47]	0.249	28.08 [23.96, 34.20]	28.65 [24.58, 34.38]	0.433
**Race (%)**			0.667			
WHITE	465 (59.2)	194 (59.7)		179 (61.7)	172 (59.3)	
ASIAN	17 (2.2)	11 (3.4)		7 (2.4)	7 (2.4)	
BLACK	95 (12.1)	37 (11.4)		39 (13.4)	35 (12.1)	
OTHER or UKNOWN	209 (26.6)	83 (25.5)		65 (22.4)	76 (26.2)	
**Admission ICU**			0.015			0.342
Medical ICU (%)	533 (67.8)	195 (60.0)		191 (65.9)	179 (61.7)	
Surgical ICU (%)	253 (32.2)	130 (40.0)		99 (34.1)	111 (38.3)	
Vital indicators						
HR (bpm)	87.44 [76.00, 99.03]	92.00 [79.48, 104.38]	<0.001	90.12 [80.10, 102.09]	90.72 [78.57, 103.12]	0.717
RR (bpm)	20.40 [17.65, 23.42]	20.95 [18.40, 24.23]	0.041	20.75 [18.16, 23.96]	20.91 [18.56, 24.11]	0.731
Temperature (°C)	37.67 [37.17, 38.28]	37.56 [37.11, 38.22]	0.261	37.61 [37.17, 38.28]	37.56 [37.17, 38.22]	0.909
MAP (mmHg)	77.88 [72.57, 84.19]	76.91 [72.14, 82.85]	0.056	76.39 [72.04, 83.64]	77.37 [72.45, 83.15]	0.547
Glucose (mg/dL)	142.25 [117.68, 183.70]	138.86 [110.75, 178.14]	0.141	144.90 [118.77, 185.38]	138.25 [113.00, 178.07]	0.105
First-day urine output (mL)	1382.50 [770.50, 2233.75]	1113.00 [455.00, 1955.00]	<0.001	1286.00 [677.00, 2106.25]	1165.50 [548.00, 2040.00]	0.129
Laboratory indicators						
PH	7.41 [7.37, 7.46]	7.40 [7.36, 7.46]	0.209	7.41 [7.35, 7.45]	7.40 [7.36, 7.45]	0.675
PO2 (mm Hg)	85.00 [69.00, 106.15]	81.00 [68.00, 102.00]	0.155	85.00 [69.00, 101.75]	82.50 [69.00, 103.00]	0.772
PCO2 (mm Hg)	45.00 [39.00, 55.00]	43.50 [37.70, 53.00]	0.035	44.55 [39.00, 53.75]	43.80 [38.00, 54.00]	0.509
Pao2/Fio2 (P/F, mmHg)	281.08 [208.33, 367.50]	286.00 [226.00, 375.00]	0.114	283.37 [212.34, 370.43]	284.93 [225.56, 374.75]	0.667
Lactate (mmol/L)	2.20 [1.50, 3.69]	2.80 [1.70, 4.60]	<0.001	2.40 [1.60, 4.27]	2.70 [1.70, 4.30]	0.247
WBC (×10^9/L)	13.95 [10.00, 19.40]	15.80 [10.20, 20.50]	0.061	15.00 [10.10, 20.80]	15.20 [10.03, 20.25]	0.780
Hemoglobin (g/dL)	11.00 [9.50, 12.80]	10.50 [9.40, 12.30]	0.042	10.70 [9.40, 12.60]	10.60 [9.40, 12.30]	0.599
Platelets (×10^9/L)	198.50 [130.00, 272.00]	176.00 [113.00, 266.00]	0.038	185.00 [113.25, 261.75]	177.00 [116.50, 266.00]	0.996
Albumin (g/dL)	3.20 [2.80, 3.60]	3.07 [2.78, 3.50]	0.029	3.20 [2.80, 3.60]	3.10 [2.80, 3.50]	0.440
BUN (mg/dL)	29.00 [18.00, 53.00]	33.00 [20.00, 55.00]	0.025	32.50 [19.00, 54.00]	33.00 [20.00, 53.00]	0.618
Creatinine (mg/dL)	1.40 [0.90, 2.50]	1.90 [1.10, 3.30]	<0.001	1.60 [0.90, 2.78]	1.90 [1.00, 3.20]	0.121
Calcium (mg/dL)	8.40 [7.90, 9.10]	8.50 [8.00, 9.00]	0.318	8.50 [8.03, 9.10]	8.40 [7.90, 9.10]	0.356
Chloride (mmol/L)	106.00 [101.00, 111.00]	105.00 [100.00, 110.00]	0.055	104.00 [99.00, 109.00]	105.00 [100.00, 111.00]	0.211
Sodium (mmol/L)	141.00 [138.00, 144.00]	140.00 [136.00, 143.00]	<0.001	140.00 [136.00, 142.75]	140.00 [136.00, 143.00]	0.621
Potassium (mmol/L)	4.40 [4.00, 5.10]	4.70 [4.20, 5.40]	<0.001	4.55 [4.10, 5.40]	4.60 [4.20, 5.27]	0.506
Medications and interventions						
Vasopressors (%)	441 (56.1)	198 (60.9)	0.158	182 (62.8)	170 (58.6)	0.350
Continuous renal replacement therapy (%)	47 (6.0)	29 (8.9)	0.102	21 (7.2)	23 (7.9)	0.875
Invasive mechanical ventilation (%)	631 (80.3)	226 (69.5)	<0.001	202 (69.7)	209 (72.1)	0.584
Invasive arterial pressure monitoring (%)	453 (57.6)	189 (58.2)	0.926	164 (56.6)	164 (56.6)	1.000
Peripherally inserted central catheter (%)	93 (11.8)	30 (9.2)	0.249	31 (10.7)	29 (10.0)	0.892
**Disease severity scoring system**						
SOFA	4.00 [2.00, 6.00]	4.00 [3.00, 6.00]	0.053	4.00 [3.00, 6.00]	4.00 [3.00, 6.00]	0.961
LODS	7.00 [5.00, 9.00]	7.00 [6.00, 10.00]	<0.001	7.00 [5.00, 9.00]	7.00 [6.00, 9.00]	0.535
OASIS	37.00 [32.00, 43.00]	39.00 [33.00, 45.00]	0.074	37.00 [32.00, 43.00]	39.00 [33.00, 43.75]	0.195
APS III	57.50 [44.00, 74.00]	66.00 [51.00, 82.00]	<0.001	63.00 [48.00, 81.00]	64.50 [50.00, 79.75]	0.774
CCI	5.00 [3.00, 7.00]	5.00 [3.00, 7.00]	0.407	5.00 [3.00, 8.00]	5.00 [3.00, 7.00]	0.698
**Comorbidities**						
Congestive heart failure (%)	232 (29.5)	93 (28.6)	0.820	80 (27.6)	84 (29.0)	0.782
Chronic pulmonary disease (%)	250 (31.8)	84 (25.8)	0.058	95 (32.8)	81 (27.9)	0.240
Mild liver disease (%)	212 (27.0)	122 (37.5)	0.001	101 (34.8)	103 (35.5)	0.931
Diabetes (%)	272 (34.6)	102 (31.4)	0.335	95 (32.8)	96 (33.1)	1.000
Renal disease (%)	186 (23.7)	92 (28.3)	0.121	79 (27.2)	78 (26.9)	1.000
Cancer (%)	82 (10.4)	39 (12.0)	0.511	36 (12.4)	36 (12.4)	1.000

EN, enteral nutrition; BMI, body mass index; MICU, medical intensive care unit; SICU, surgical intensive care unit; HR, heart rate; RR, respiratory rate; MAP, mean arterial pressure; PH, potential of hydrogen; PO2, partial pressure of oxygen; PCO2, partial pressure of carbon dioxide; WBC, white blood cell; BUN, blood urea nitrogen; SOFA, sequential organ failure assessment; LODS, logistic organ dysfunction system; OASIS, oxford acute severity of illness score; APS III, acute physiology score III; CCI, Charlson comorbidity index.

### 3.2 Comparison of the primary outcome before and after PSM

**Figure 2:**
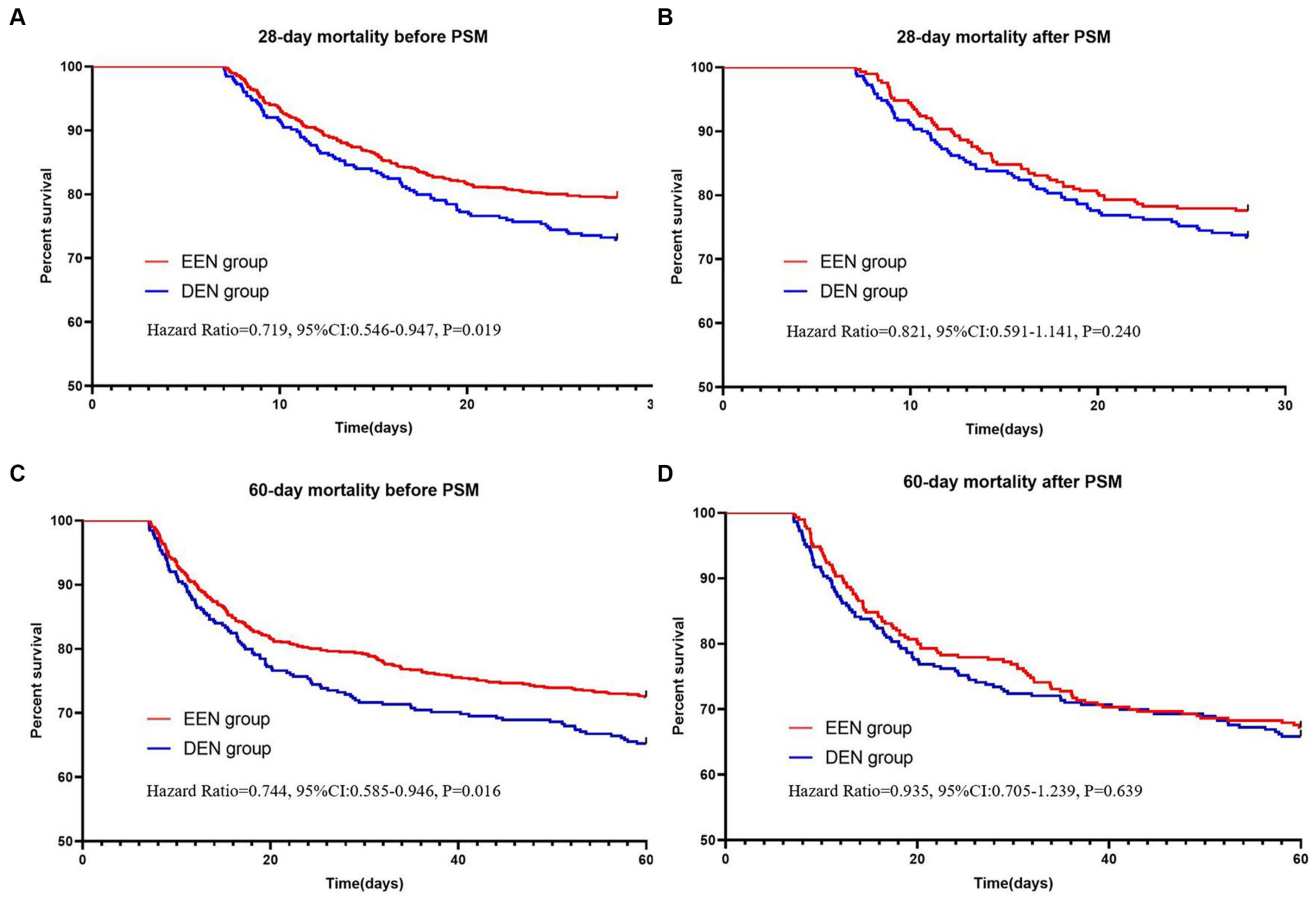
Kaplan–Meier survival curves of the two groups at 28 **(A,B)** and 60 **(C,D)** days before and after propensity score matching.

Before propensity score matching (PSM), 28-day mortality was significantly lower in the early enteral nutrition group compared to the delayed enteral nutrition group (161 (20.5%) vs. 88 (27.1%); *p* = 0.018) (Table 2). The univariate Kaplan–Meier survival curve for 28 days also indicated that the early enteral nutrition group had a longer survival time (HR = 0.719, 95% CI: 0.546–0.947; *p* = 0.019) (Figure 2A). After PSM, all standardized mean differences were less than 0.1, indicating similar distributions of baseline variables in both groups (Supplementary Table S1). Following propensity matching, 28-day mortality was 4.2% lower in the early enteral nutrition group compared to the delayed enteral nutrition group, but this difference was not statistically significant (65 (22.4%) vs. 77 (26.6%); *p* = 0.247). The 28-day Kaplan–Meier curve post-propensity matching echoed the propensity-matched result (HR = 0.821, 95% CI: 0.591–1.141, *p* = 0.240) (Figure 2B). Additionally, we conducted multifactor logistic regression analysis to validate the results of propensity matching, incorporating age, gender, and covariates with *p*-values <0.05. The results of the multifactor logistic analysis (OR = 0.778 (0.559–1.084); *p* = 0.138) were consistent with those of propensity matching. Propensity matching suggested that early enteral nutrition did not significantly reduce 28-day mortality in sepsis patients compared with delayed enteral nutrition.

**Table 2:** Primary and secondary outcomes.

Outcomes	Patients before PSM (*n* = 1,111)			Patients after PSM (*n* = 580)		
	Early EN (*n* = 786)	Delayed EN (*n* = 325)	*p*-value	Early EN (*n* = 290)	Delayed EN (*n* = 290)	*p-*value
28-day mortality (%)	161 (20.5)	88 (27.1)	0.018	65 (22.4)	77 (26.6)	0.247
60-day mortality (%)	216 (27.5)	113 (34.8)	0.017	95 (32.8)	99 (34.1)	0.725
ICU mortality (%)	97 (12.3)	45 (13.8)	0.491	41 (14.1)	39 (13.4)	0.810
ICU LOS (days)	8.3 [5.2,12.3]	10.0 [7.5–14.2]	<0.001	8.3 [5.2,12.4]	10.1 [7.5,14.2]	<0.001
AKI stage (%)			<0.001			0.030
Stage 0	70 (8.9)	10 (3.1)		26 (9.0)	10 (3.4)	
Stage 1	103 (13.1)	25 (7.7)		30 (10.3)	24 (8.3)	
Stage 2	280 (35.6)	106 (32.6)		91 (31.4)	95 (32.8)	
Stage 3	333 (42.4)	184 (56.6)		143 (49.3)	161 (55.5)	

ICU, intensive care unit; LOS, length of stay; AKI, acute kidney injury.

### 3.3 Comparison of secondary outcomes before and after PSM

**Figure 3:**
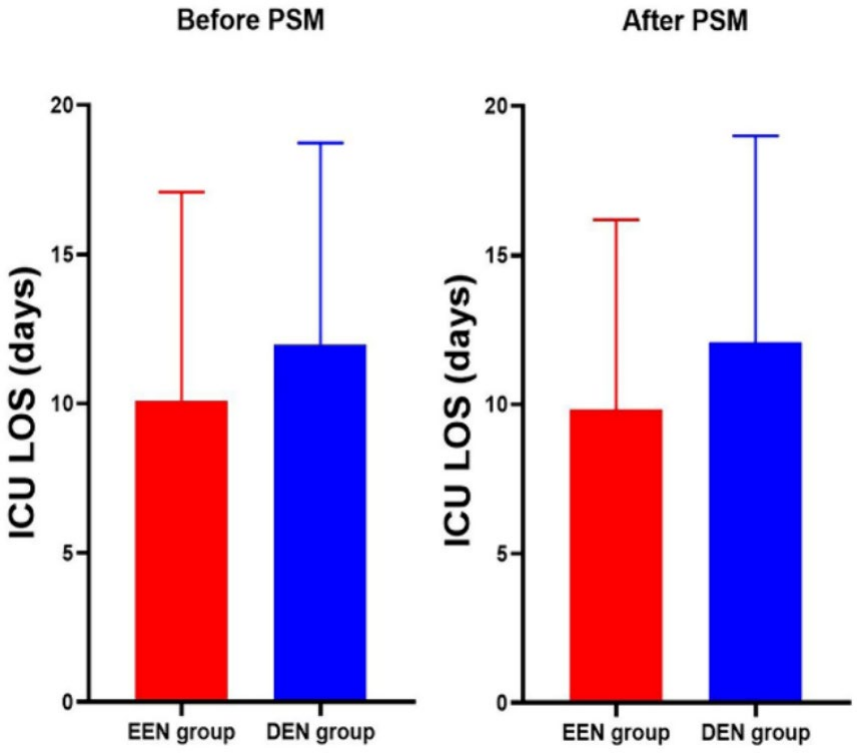
ICU length of stay before and after propensity score matching.

**Figure 4:**
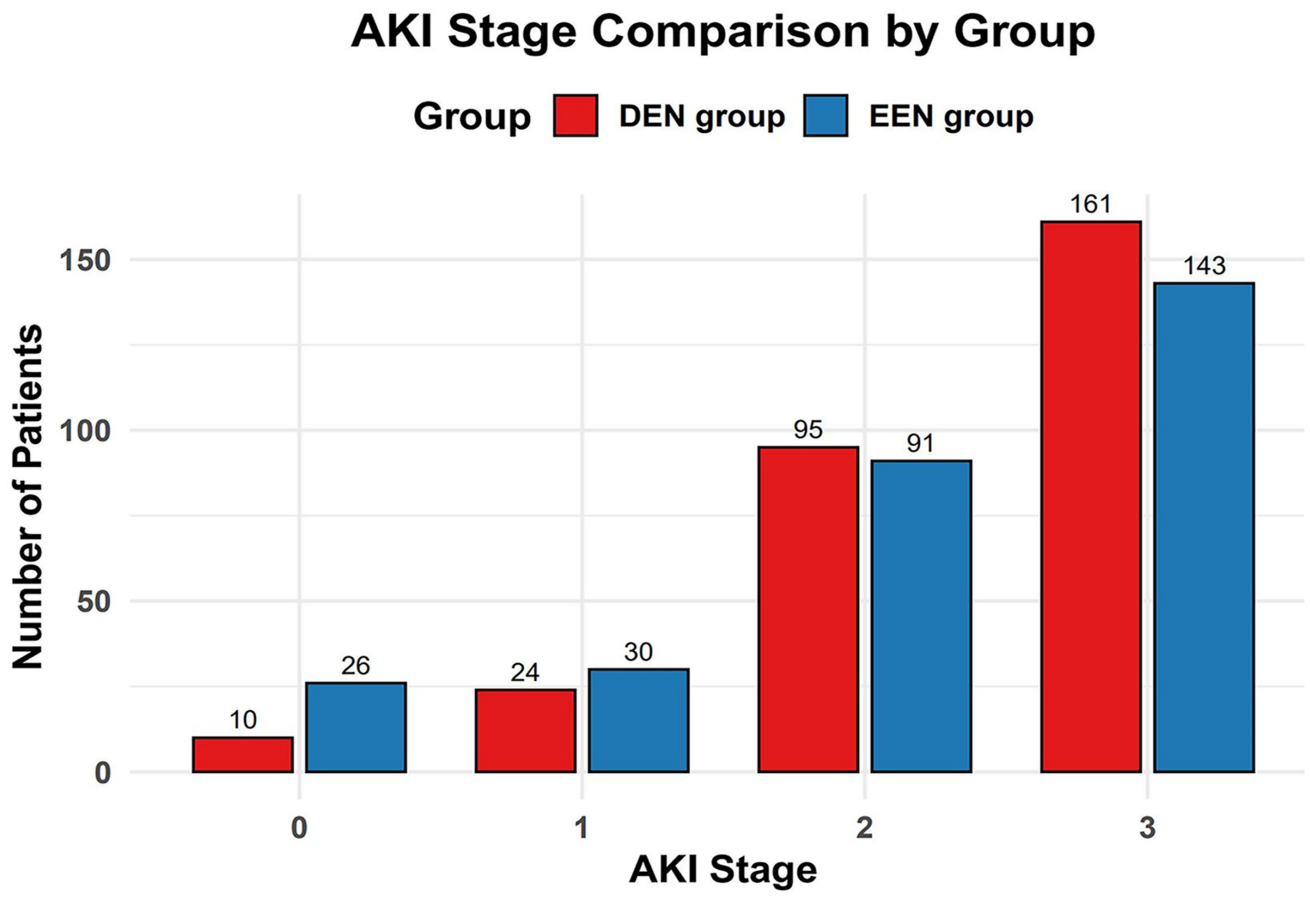
Comparison of AKI stage incidence between the early and delayed groups after propensity score matching.

Before propensity matching, 60-day mortality was lower in the early enteral nutrition group than in the late enteral nutrition group (216 (27.5) vs. 113 (34.8); *p* = 0.017) (Table 2). The univariate Kaplan–Meier survival curve also showed longer survival in the early enteral nutrition group (HR = 0.744, 95% CI: 0.585–0.946, *p* = 0.017) (Figure 2C). The EEN group had shorter length of ICU stay (8.3 [5.2, 12.3] vs. 10.0 [7.5–14.2]; *p* < 0.001) and a lower incidence of stage 3 AKI (333 (42.4) vs. 184 (56.6); *p* < 0.001). After propensity matching, we found no statistically significant differences in 60-day mortality (95 (32.8) vs. 99 (34.1); *p* = 0.725) between the EEN and DEN groups. The 60-day Kaplan–Meier curve after propensity matching was consistent with the result after propensity matching (hazard ratio = 0.935, 95% CI: 0.705–1.239, *p* = 0.639) (Figure 2D). Multivariate logistic analysis was performed to verify the results of propensity matching and found that 60-day mortality (aOR = 0.859, 95% CI: 0.629–1.173, *p* = 0.339) in both groups were consistent with the post-PSM results. However, the EEN group still had a shorter length of ICU stay (8.3 [5.2, 12.4] vs. 110.1 [7.5, 14.2]; *p* < 0.001) (Figure 3) and a lower incidence of severe kidney injury (143 (49.3) vs. 161 (55.5); *p* = 0.030) than the DEN group after propensity matching (Figure 4).

### 3.4 Additional analysis

**Figure 5:**
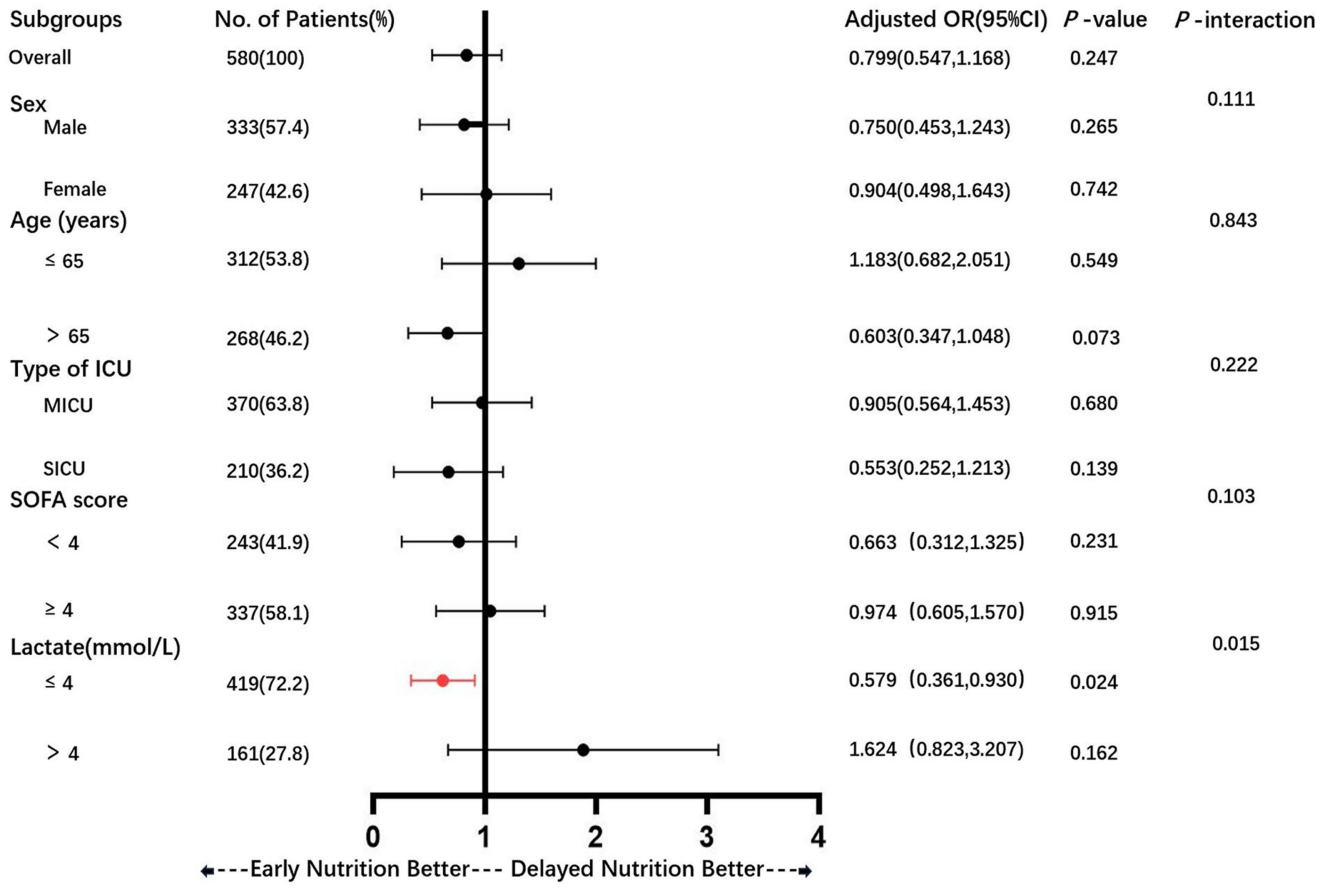
Subgroup analyses to identify the specific benefit population.

**Figure 6:**
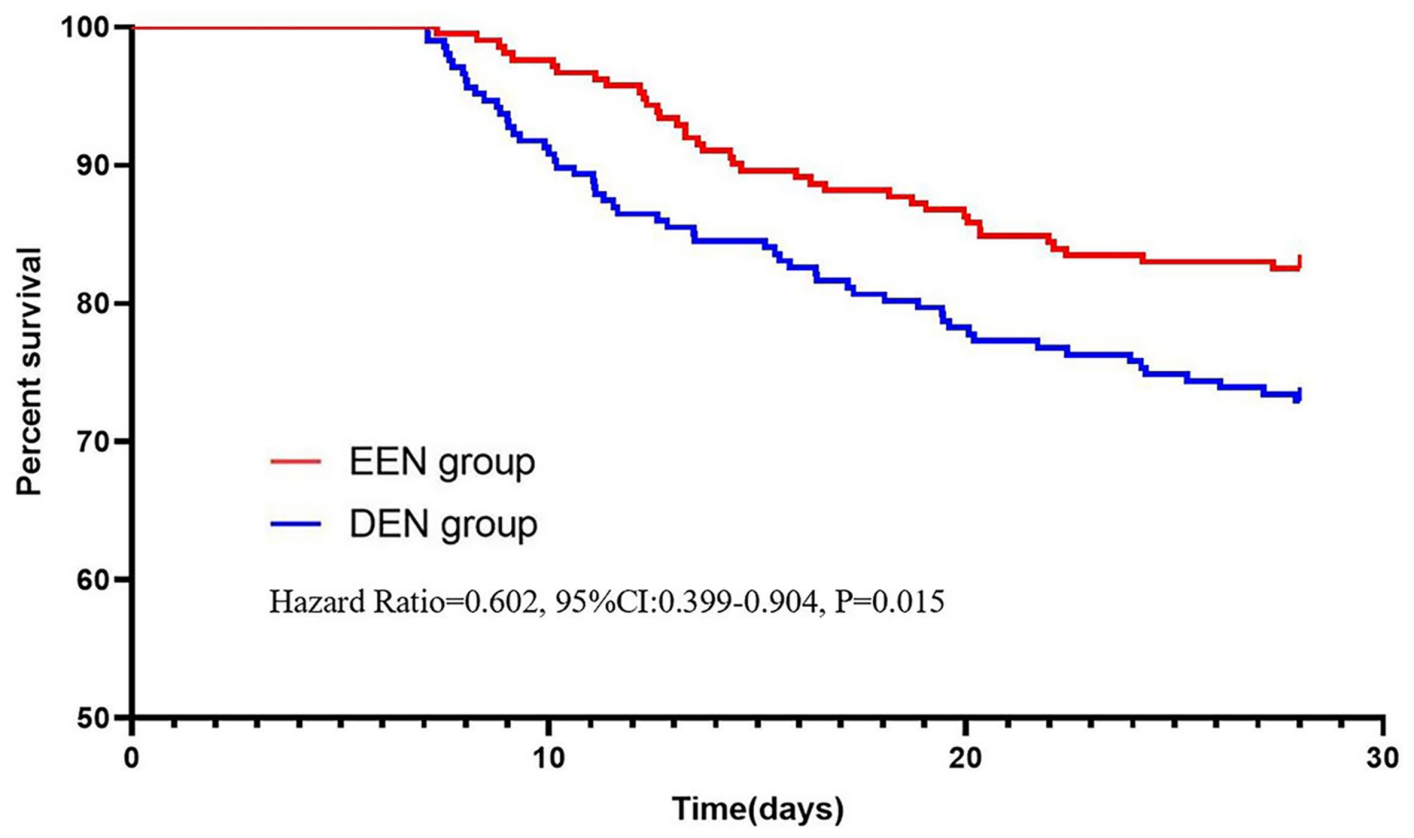
The Kaplan–Meier survival curve adjusted for the subgroup with lactate ≤4 mmol/L.

We conducted several subgroup analyses based on propensity-matched data to explore the relationship between early enteral nutrition and 28-day mortality across different subgroups of sepsis patients. Patients were categorized based on sex, age, ICU type, SOFA score, and lactate levels. Single-factor logistic analysis and multifactor logistic analysis were utilized to assess the association between early enteral nutrition and 28-day mortality within various subgroups. The results for all subgroups are illustrated in a forest plot (Figure 5). Our findings indicate that early enteral nutrition decreased the 28-day mortality rate in patients with lactate levels ≤4 mmol/L (aOR = 0.579, 95% CI: 0.361–0.930, *p* = 0.024). The COX survival analysis-adjusted Kaplan–Meier survival curve is depicted in Figure 6.

## 4 Discussion

The present study compared the outcomes between early and delayed EN in sepsis patients by propensity score matching based on the MIMIC IV 2.2 database. The results showed that early EN was not associated with mortality reduction but was associated with a lower incidence of severe acute kidney injury and a shorter length of ICU stay. This finding may reflect the regulatory effects of early enteral nutrition on inflammation and tissue damage. By providing nutritional support and improving intestinal mucosal barrier function, early enteral nutrition may alleviate systemic inflammatory responses and reduce the risk of renal injury. Additionally, early enteral nutrition may enhance hemodynamic stability and tissue perfusion, thereby minimizing renal damage. Overall, early enteral nutrition may reduce the incidence of stage 3 acute kidney injury through multiple pathways.

In the present study, we did not observe a reduction in mortality associated with early EN. This finding is consistent with previous studies in patients with sepsis and unselected critically ill patients (34–37). However, a recent study in critically ill patients found that early nutritional support in the ICU was significantly associated with increased mortality at 28 days, particularly in younger patients with less severe disease (21). However, many studies have shown the benefits of early enteral nutrition in the treatment of patients with many diseases (17, 21, 38). From a physiological point of view, early enteral nutrition therapy is potentially beneficial for patients with sepsis. Studies have shown that early enteral nutrition can maintain intestinal integrity and prevent intestinal permeability, thereby dampening the inflammatory response (20). Therefore, we performed subgroup analyses of the matched data to explore which sepsis patients would benefit from early enteral nutrition. We found that early enteral nutrition reduced 28-day mortality in the lactate ≤4 mmol/L groups (Figure 4). This observation is consistent with previous research, indicating the prognostic significance of lactate levels in sepsis. Multiple studies have demonstrated that lower lactate levels at the onset of sepsis are associated with better outcomes, including reduced mortality rates. Considering this established relationship, patients with initially lower lactate levels may represent a subgroup with less severe disease, and thus may benefit more from interventions such as early enteral nutrition. Our study further demonstrates that early enteral nutrition may have more pronounced benefits for patients with initially lower lactate levels, providing valuable insights for personalized treatment strategies. However, due to the complexity and severity of sepsis, the effect of enteral nutrition may not be obvious. Many internal factors influence the early enteral nutrition management of critically ill patients. For example, the performance of enteral nutrition in patients is affected by the use of large doses of catecholamines or the occurrence of vomiting, diarrhea, etc. Therefore, early enteral nutrition is not appropriate for all patients with sepsis (39).

Different sepsis patients require varying nutritional regimens. Our study indicates the necessity to optimize enteral nutrition in sepsis patients in the future, and appropriate nutritional intervention for different critically ill populations is crucial (40). In the treatment of sepsis through nutrition, it is necessary to rigorously identify individuals who are at risk of malnutrition (41). Individualized nutritional therapy represents a promising future direction for enteral nutrition therapy in sepsis patients.

There are several limitations to our study. First, to minimize possible confounding factors, we used propensity score matching. However, it may reduce the sample size of our study population. Although all patients in the EEN were matched and balance properties were satisfied, the distribution of the matched dataset was less comparable to the original dataset. Second, although we performed propensity score matching to control for confounding, some residual confounders may not be measured in this study. The study did not account for total caloric intake, progression of caloric intake, interruption of EN, baseline nutritional assessment, or intervention measures before enteral nutrition. These factors could have led to unmeasured confounding (42). Third, because of complex therapeutic interventions and subjective clinician decisions, we could not clearly explore the causal relationship between early EN and 28-day mortality. Finally, because the data we based on are from an observational database, the results reported in our study should be regarded only as a reference and must be further verified. Additional high-quality and larger sample size randomized trials are needed to investigate the optimal combination time for fluid administration and the optimal strategy to guide fluid therapy.

## 5 Conclusion

In conclusion, our retrospective study suggests that early enteral nutrition may not affect mortality rates when analyzed using propensity score matching. However, our findings indicate that early enteral nutrition is associated with shorter ICU stays and a lower incidence of severe acute kidney injury. Notably, subgroup analysis indicates that septic patients with lower lactate levels may derive greater benefit from early enteral nutrition. Considering the potential limitations of the propensity score method, additional randomized trials are necessary to validate the benefits of early enteral nutrition.

## Data availability statement

The datasets presented in this study can be found in online repositories. The names of the repository/repositories and accession number(s) can be found at: https://physionet.org/content/mimiciv/2.2/.

## Ethics statement

MIMIC-IV database is approved by the Massachusetts Institute of Technology Institutional Review Boards. All the patients of the database are de-identified for privacy protection and informed consent is waived by the Nanjing Drum Tower Hospital Ethics Committee. The study followed the principles of the Declaration of Helsinki.

## Author contributions

FX: Data curation, Writing – original draft. JX: Data curation, Writing – review & editing. JM: Data curation, Writing – original draft. WX: Writing – original draft. SG: Writing – review & editing. GL: Writing – review & editing. JW: Writing – review & editing.

## Glossary

**Table:** 

EN	Enteral nutrition
EEN	Early enteral nutrition
DEN	Delayed enteral nutrition
ICU	Intensive care unit
MIMIC-IV	MIMIC-IV Medical Information Mart for Intensive Care IV
PSM	Propensity score matching
SMD	Standardized mean differences
OR	Odds ratio
BMI	Body mass index
MICU	Medical intensive care unit
SICU	Surgical intensive care unit
HR	Heart rate
RR	Respiratory rate
MAP	Mean arterial pressure
PH	Potential of hydrogen
PO2	Partial pressure of oxygen
PCO2	Partial pressure of carbon dioxide
WBC	White blood cell
BUN	Blood urea nitrogen
SOFA	Sequential Organ Failure Assessment
LODS	Logistic Organ Dysfunction System
OASIS	Oxford Acute Severity of Illness Score
APS III	Acute Physiology Score III
CCI	Charlson Comorbidity Index
LOS	Length of Stay
AKI	Acute kidney injury

## Footnotes

^1^ https://github.com/MIT-LCP/mimic-iv
