# Supplementary material for: Early versus delayed enteral nutrition in ICU patients with sepsis: a propensity score-matched analysis based on the MIMIC-IV database
Source: Front Nutr. 2024 Jun 24;11:1370472. doi: 10.3389/fnut.2024.1370472 (PMC11228309; doi:10.3389/fnut.2024.1370472)
Supplement: Supplementary file 1 [file Table_1.DOCX]

**Additional Table 1**: Demographic data and comparisons between the early EN group and the delayed EN group before and after matching

|  | **Before PSM** | |  |  | **After PSM** | |  |  |
| --- | --- | --- | --- | --- | --- | --- | --- | --- |
| **Variables** | **Early EN（n=786）** | **Delayed EN（n=325）** | ***p*-value** | **SMD** | **Early EN（n=290）** | **Delayed EN（n=290）** | ***p*-value** | **SMD** |
| **Age(years)** | 64.23 [52.88, 74.45] | 62.30 [51.62, 73.59] | 0.198 | 0.076 | 64.13 [51.76, 73.08] | 63.27 [52.16, 73.94] | 0.908 | 0.021 |
| **Male** | 412 (52.4) | 191 (58.8) | 0.062 | 0.128 | 169(58.3) | 164(56.6) | 0.737 | 0.035 |
| **BMI(kg/m2)** | 28.10 [24.26, 34.03] | 28.83 [24.56, 34.47] | 0.249 | 0.061 | 28.08 [23.96, 34.20] | 28.65 [24.58, 34.38] | 0.433 | 0.003 |
| **Race (%)** |  |  | 0.667 |  |  |  |  |  |
| WHITE | 465(59.2) | 194(59.7) |  | 0.011 | 179(61.7) | 172(59.3) |  | 0.049 |
| ASIAN | 17(2.2) | 11(3.4) |  | 0.074 | 7(2.4) | 7(2.4) |  | <0.001 |
| BLACK | 95(12.1) | 37(11.4) |  | 0.022 | 39(13.4) | 35(12.1) |  | 0.041 |
| OTHER or UKNOWN | 209(26.6) | 83(25.5) |  | 0.024 | 65(22.4) | 76(26.2) |  | 0.089 |
| **Admission ICU** |  |  | 0.015 |  |  |  | 0.342 |  |
| Medical ICU (%) | 533(67.8) | 195(60.0) |  | 0.163 | 191(65.9) | 179(61.7) |  | 0.086 |
| Surgical ICU(%) | 253(32.2) | 130(40.0) |  | 0.163 | 99(34.1) | 111(38.3) |  | 0.086 |
| **Vital Indicators** |  |  |  |  |  |  |  |  |
| HR（bpm) | 87.44 [76.00, 99.03] | 92.00 [79.48, 104.38] | <0.001 | 0.278 | 90.12 [80.10, 102.09] | 90.72 [78.57, 103.12] | 0.717 | 0.055 |
| RR (bpm) | 20.40 [17.65, 23.42] | 20.95 [18.40, 24.23] | 0.041 | 0.110 | 20.75 [18.16, 23.96] | 20.91 [18.56, 24.11] | 0.731 | 0.015 |
| Temperature (°C) | 37.67 [37.17, 38.28] | 37.56 [37.11, 38.22] | 0.261 | 0.061 | 37.61 [37.17, 38.28] | 37.56 [37.17, 38.22] | 0.909 | 0.014 |
| MAP(mmHg) | 77.88 [72.57, 84.19] | 76.91 [72.14, 82.85] | 0.056 | 0.124 | 76.39 [72.04, 83.64] | 77.37 [72.45, 83.15] | 0.547 | 0.014 |
| Glucose (mmol/L) | 142.25 [117.68, 183.70] | 138.86 [110.75, 178.14] | 0.141 | 0.035 | 144.90 [118.77, 185.38] | 138.25 [113.00, 178.07] | 0.105 | 0.006 |
| First-day Urine Output (mL) | 1382.50 [770.50, 2233.75] | 1113.00 [455.00, 1955.00] | <0.001 | 0.176 | 1286.00 [677.00, 2106.25] | 1165.50 [548.00, 2040.00] | 0.129 | 0.09 |
| **Laboratory Indicators** |  |  |  |  |  |  |  |  |
| PH | 7.41 [7.37, 7.46] | 7.40 [7.36, 7.46] | 0.209 | 0.089 | 7.41 [7.35, 7.45] | 7.40 [7.36, 7.45] | 0.675 | 0.035 |
| PO2 (mm Hg) | 85.00 [69.00, 106.15] | 81.00 [68.00, 102.00] | 0.155 | 0.083 | 85.00 [69.00, 101.75] | 82.50 [69.00, 103.00] | 0.772 | 0.051 |
| PCO2 (mm Hg) | 45.00 [39.00, 55.00] | 43.50 [37.70, 53.00] | 0.035 | 0.155 | 44.55 [39.00, 53.75] | 43.80 [38.00, 54.00] | 0.509 | 0.031 |
| Pao2/Fio2（P/F, mmHg） | 281.08 [208.33, 367.50] | 286.00 [226.00, 375.00] | 0.114 | 0.160 | 283.37 [212.34, 370.43] | 284.93 [225.56, 374.75] | 0.667 | 0.054 |
| Lactate(mmol/L) | 2.20 [1.50, 3.69] | 2.80 [1.70, 4.60] | <0.001 | 0.188 | 2.40 [1.60, 4.27] | 2.70 [1.70, 4.30] | 0.247 | 0.036 |
| WBC (×10^9/L） | 13.95 [10.00, 19.40] | 15.80 [10.20, 20.50] | 0.061 | 0.107 | 15.00 [10.10, 20.80] | 15.20 [10.03, 20.25] | 0.780 | 0.038 |
| Hemoglobin (g/dL) | 11.00 [9.50, 12.80] | 10.50 [9.40, 12.30] | 0.042 | 0.124 | 10.70 [9.40, 12.60] | 10.60 [9.40, 12.30] | 0.599 | 0.031 |
| Platelets (×10^9/L) | 198.50 [130.00, 272.00] | 176.00 [113.00, 266.00] | 0.038 | 0.118 | 185.00 [113.25, 261.75] | 177.00 [116.50, 266.00] | 0.996 | 0.005 |
| Albumin (g/dL) | 3.20 [2.80, 3.60] | 3.07 [2.78, 3.50] | 0.029 | 0.119 | 3.20 [2.80, 3.60] | 3.10 [2.80, 3.50] | 0.440 | 0.051 |
| BUN (mg/dL) | 29.00 [18.00, 53.00] | 33.00 [20.00, 55.00] | 0.025 | 0.089 | 32.50 [19.00, 54.00] | 33.00 [20.00, 53.00] | 0.618 | 0.041 |
| Creatinine (mg/dL) | 1.40 [0.90, 2.50] | 1.90 [1.10, 3.30] | <0.001 | 0.118 | 1.60 [0.90, 2.78] | 1.90 [1.00, 3.20] | 0.121 | 0.016 |
| Calcium (mg/dL) | 8.40 [7.90, 9.10] | 8.50 [8.00, 9.00] | 0.318 | 0.027 | 8.50 [8.03, 9.10] | 8.40 [7.90, 9.10] | 0.356 | 0.034 |
| Chloride (mmol/L) | 106.00 [101.00, 111.00] | 105.00 [100.00, 110.00] | 0.055 | 0.147 | 104.00 [99.00, 109.00] | 105.00 [100.00, 111.00] | 0.211 | 0.073 |
| Sodium (mmol/L) | 141.00 [138.00, 144.00] | 140.00 [136.00, 143.00] | <0.001 | 0.229 | 140.00 [136.00, 142.75] | 140.00 [136.00, 143.00] | 0.621 | 0.027 |
| Potassium (mmol/L) | 4.40 [4.00, 5.10] | 4.70 [4.20, 5.40] | <0.001 | 0.205 | 4.55 [4.10, 5.40] | 4.60 [4.20, 5.27] | 0.506 | 0.033 |
| **Medications and interventions** |  |  |  |  |  |  |  |  |
| Vasopressors (%) | 441(56.1) | 198(60.9) | 0.158 | 0.098 | 182(62.8) | 170(58.6) | 0.350 | 0.085 |
| Continuous renal replacement therapy (%) | 47(6.0) | 29(8.9) | 0.102 | 0.112 | 21(7.2) | 23(7.9) | 0.875 | 0.026 |
| Invasive mechanical ventilation(%) | 631(80.3) | 226(69.5) | <0.001 | 0.250 | 202(69.7) | 209(72.1) | 0.584 | 0.053 |
| Invasive arterial pressure monitoring (%) | 453(57.6) | 189(58.2) | 0.926 | 0.011 | 164(56.6) | 164(56.6) | 1.000 | <0.001 |
| Peripherally inserted central catheter (%) | 93(11.8) | 30(9.2) | 0.249 | 0.085 | 31(10.7) | 29(10.0) | 0.892 | 0.023 |
| **Disease severity scoring system** |  |  |  |  |  |  |  |  |
| SOFA | 4.00 [2.00, 6.00] | 4.00 [3.00, 6.00] | 0.053 | 0.118 | 4.00 [3.00, 6.00] | 4.00 [3.00, 6.00] | 0.961 | 0.009 |
| LODS | 7.00 [5.00, 9.00] | 7.00 [6.00, 10.00] | <0.001 | 0.118 | 7.00 [5.00, 9.00] | 7.00 [6.00, 9.00] | 0.535 | 0.037 |
| OASIS | 37.00 [32.00, 43.00] | 39.00 [33.00, 45.00] | 0.074 | 0.099 | 37.00 [32.00, 43.00] | 39.00 [33.00, 43.75] | 0.195 | 0.078 |
| APS III | 57.50 [44.00, 74.00] | 66.00 [51.00, 82.00] | <0.001 | 0.327 | 63.00 [48.00, 81.00] | 64.50 [50.00, 79.75] | 0.774 | 0.010 |
| CCI | 5.00 [3.00, 7.00] | 5.00 [3.00, 7.00] | 0.407 | 0.073 | 5.00 [3.00, 8.00] | 5.00 [3.00, 7.00] | 0.698 | 0.021 |
| **Comorbidities** |  |  |  |  |  |  |  |  |
| Congestive heart failure (%) | 232(29.5) | 93(28.6) | 0.820 | 0.020 | 80(27.6) | 84(29.0) | 0.782 | 0.031 |
| Chronic pulmonary disease (%) | 250(31.8) | 84(25.8) | 0.058 | 0.132 | 95(32.8) | 81(27.9) | 0.240 | 0.105 |
| Mild liver disease (%) | 212(27.0) | 122(37.5) | 0.001 | 0.228 | 101(34.8) | 103(35.5) | 0.931 | 0.014 |
| Diabetes (%) | 272(34.6) | 102(31.4) | 0.335 | 0.069 | 95(32.8) | 96(33.1) | 1.000 | 0.007 |
| Renal disease (%) | 186(23.7) | 92(28.3) | 0.121 | 0.106 | 79(27.2) | 78(26.9) | 1.000 | 0.008 |
| Cancer (%) | 82(10.4) | 39(12.0) | 0.511 | 0.050 | 36(12.4) | 36(12.4) | 1.000 | <0.001 |
