# Supplementary figures and images for: Early versus delayed enteral nutrition in ICU patients with sepsis: a propensity score-matched analysis based on the MIMIC-IV database
Source: Front Nutr. 2024 Jun 24;11:1370472. doi: 10.3389/fnut.2024.1370472 (PMC11228309; doi:10.3389/fnut.2024.1370472)

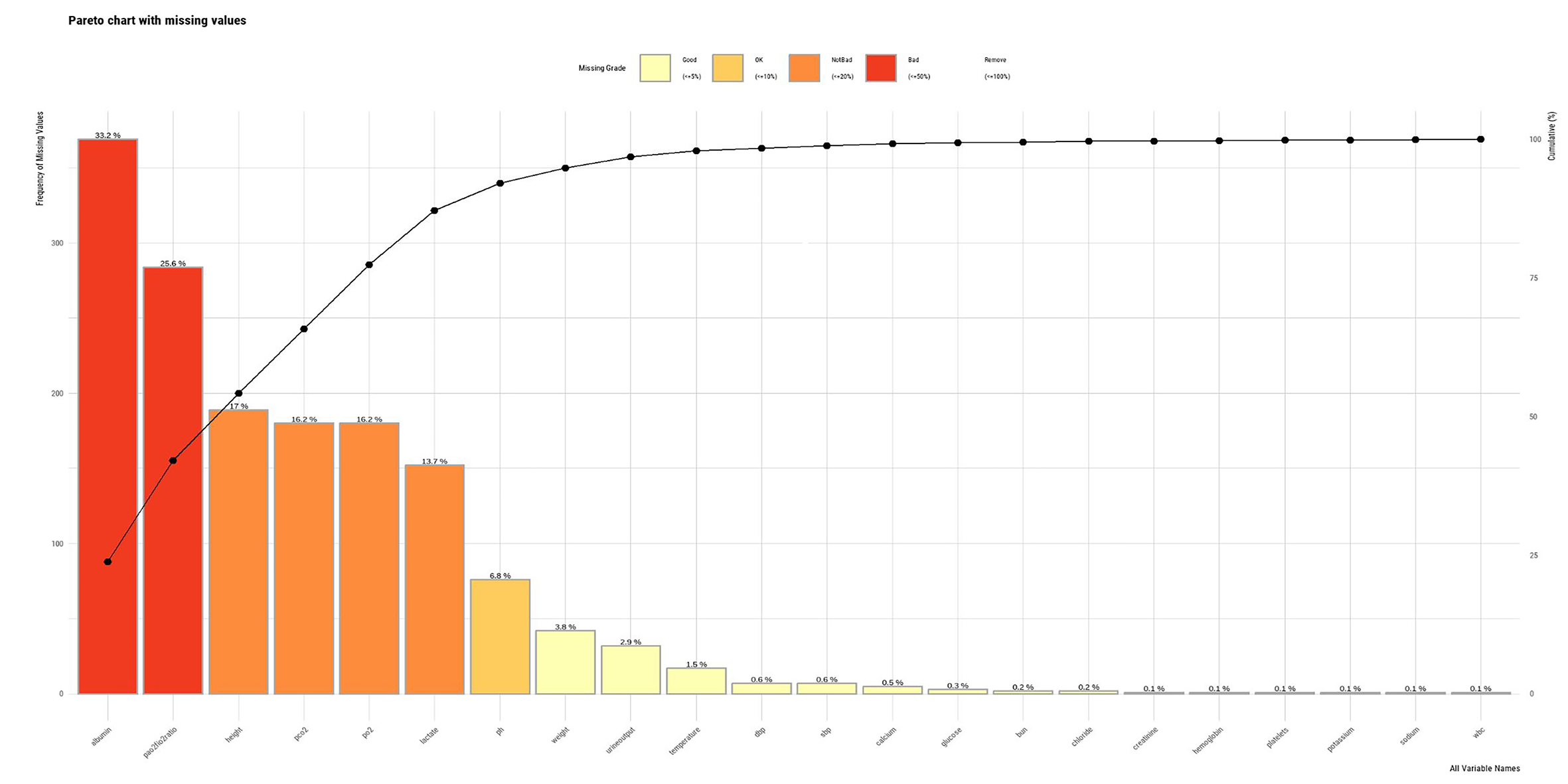

Supplement: Supplementary file 2 [file Image_1.tif]
